# Transmission of SARS-CoV-2 among underserved pastoralist communities in Kajiado County, Kenya: 2020–2022

**DOI:** 10.1371/journal.pone.0308318

**Published:** 2024-08-08

**Authors:** Zipporah Macharia, Brian Ogoti, Magdaline Otieno, Pauline Gitonga, Angela Bosco-Lauth, Marybeth Maritim, Esther Lemarkoko, Aggrey Keya, Joseph Sankok, George Gitao, Joshua Onono, Julius Oyugi, Richard A. Bowen

**Affiliations:** 1 Institute of Tropical and Infectious Diseases (UNITID), University of Nairobi, Nairobi, Kenya; 2 Department of Medical Microbiology and Immunology, University of Nairobi, Nairobi, Kenya; 3 Center of Epidemiological Modelling and Analysis, University of Nairobi, Nairobi, Kenya; 4 Department of Biomedical Sciences, Colorado State University, Fort Collins, Colorado, United States of America; 5 Department of Clinical Medicine and Therapeutics, University of Nairobi, Nairobi, Kenya; 6 Kajiado County Referral Hospital, Kajiado Town, Kajiado, Kenya; 7 Department of Pathology, Microbiology and Parasitology, University of Nairobi, Nairobi, Kenya; 8 Department of Public Health Pharmacology and Toxicology, University of Nairobi, Nairobi, Kenya; University of Ilorin, NIGERIA

## Abstract

Initial transmission of severe acute respiratory syndrome virus-2 (SARS-CoV-2) was highest in densely populated regions of Kenya. Transmission gradually trickled down to the less densely populated, remote and underserved regions such as the pastoral regions of Kajiado County which are characterized by poor healthcare systems. Molecular assays that were pivotal for COVID-19 diagnosis were not available in these regions. Serology is an alternative method for retrospectively tracking the transmission of SARS-CoV-2 in such populations. Dry blood spots (DBS) were prepared from consenting patients attending six health facilities in Kajiado County from March 2020 to March 2022. Upon elution, we conducted an enzyme-linked immunosorbent assay (ELISA) for the detection of SARS-Cov-2 IgG antibodies. Of the 908 DBSs we analyzed, 706 (78%) were from female participants. The overall seropositivity to SARS-Cov-2 antibodies was 7.3% (95% CI 5.7–9.1). The elderly (over 60 years) and male participants had a high likelihood of testing positive for SAR-CoV-2 infections. Mashuru (15.6%, 14/90) and Meto (15%, 19/127) health facilities registered the highest proportion of seropositive participants. Evidence of SARS-CoV-2 transmission among pastoralists in the remote and underserved regions of Kajiado County was established by DBS sampling and serologic testing.

## Introduction

The family Coronaviridae consists of 4 genera; alpha coronavirus, beta coronavirus, gamma coronavirus and delta Coronavirus [1]. Of the four genera, the beta coronaviruses; severe acute respiratory syndrome virus (SARS-CoV), Middle East respiratory syndrome virus (MERS-CoV), and severe acute respiratory syndrome virus-2 (SARS- CoV-2) are the most virulent to man and have been implicated in notable outbreaks in the last twenty years [1, 2]. Severe acute respiratory syndrome virus-2 is the etiologic agent of the coronavirus disease of 2019 (COVID-19), a novel and emerging infectious disease that was declared a pandemic by the World Health Organization (WHO) on March 11, 2020 [3].

After the first case of COVID-19 was identified in Wuhan, China, rapid transmission followed across the world and by November 3, 2022, more than 663 million confirmed cases and about 6.7 million fatalities had been documented globally [WHO]. As of January, 2023, more than 8.9 million cases of COVID-19 had been confirmed in Africa, with approximately 174,000 deaths [WHO]. North America, Europe and Asia bore the highest burden of the pandemic [4–6]. Kenya confirmed the first case of COVID-19 on March 13, 2020, and by November 9, 2022, 339,981 cases and 5678 deaths had been confirmed (Kenya, MOH 2022). Although transmission of SARS-CoV-2 was thought to be primarily zoonotic, it seems clear that the pandemic resulted from highly efficient human to human transmission [7, 8]. Moreover, anthropogenic activities such as travel and trade were evidenced as the greatest contributors to the spread of SARS-CoV-2 globally [7, 9, 10].

The initial spread of the original parental lineage of SARS-CoV-2 from Wuhan, China, was soon followed by the emergence of various waves of COVID-19 across the globe [11]. These waves could have been attributed to high viral transmission due to little or no adherence to mitigation measures, declining immunity after initial infection and inception of new SARS-CoV-2 variants [8, 12, 13]. SARS-CoV-2, being an RNA virus, has a high propensity to mutate into variants of interest (VOI), variants of concern (VOC) or variants of high consequence (VOHC) [14]. Examples of VOI include Zeta (Brazil), Lambda (Peru), Lota (New York), Kappa (India), Eta (UK and Nigeria), Epsilon (California, USA) and Theta variants (Philippines and Japan). However, these variants had limited global spread. Conversely, major waves were linked to VOCs; the Alpha variant which was first identified in the United Kingdom (UK), Beta variant (South Africa), Delta variant (India), Gamma variant (Brazil), and Omicron variant (South Africa) likely due to the variants increased transmissibility, infectivity, poor responsiveness to medication, resistance to immune antibodies and vaccines and severe disease outcomes [11, 13–15].

During the time frame of this study, Kenya experienced five waves of COVID-19 since the confirmation of the first case in March 2020. The first wave occurred between August and September 2020, etiologically linked to the B.1 global parental lineage of SARS-CoV-2, followed by a second wave between November 2020 and January 2021 with Beta and Alpha variants taking preeminence. Between January 2021 and February 2022, three waves erupted in the country [13]. The third wave was marked by traces of Beta variant and dominance of the Alpha variant while the fourth and the fifth waves were marked by the Delta and Omicron variants respectively [11, 13]. The last wave waned off slowly, until November 2022 when a few new cases of COVID-19 were again reported by the Ministry of Health [MOH-Kenya 2022].

At inception of SARS-CoV-2 transmission in Kenya, the government through the MOH instituted strict policy measures to reduce the spread of the virus, which included termination of international travel, prohibition of social gatherings and cessation of learning and worship. In addition, there was compulsory wearing of masks, social distancing, and restricted movements [11, 12]. Strict enforcement of disease control measures and surveillance were concentrated in the urban regions of Nairobi and Mombasa due to the perceived risk of heightened SARS-CoV-2 transmission in densely populated regions [16]. On the contrary, the less densely populated rural regions especially the remotely located pastoralist areas were disadvantaged with regard to diagnostic and surveillance monitoring [17]. This is evidenced by the paucity of published data on COVID-19 burden in remote regions of Kenya and in Africa in general [18].

Effective control, management and surveillance of COVID-19 requires prompt and reliable laboratory diagnosis to inform policy decisions [8]. Real time reverse transcriptase-polymerase chain reaction (RT-PCR) was deemed the gold standard for diagnosis and surveillance of COVID-19 across the world [19]. Albeit, this technique is costly and requires biosafety level 2 containment, specialized laboratory equipment and highly skilled personnel which largely precluded its use in underserved pastoralist communities [20, 21]. These challenges in large parts of Africa were further compounded by the already struggling economies and stressed healthcare systems [4]. Alternative low-cost serological assays would have alleviated the burden and the demand for COVID-19 diagnosis and surveillance [21].

Serological assays which have high specificity and sensitivity are technically simple, affordable and can complement RT-PCR. These assays have been of value in surveillance, epidemiological investigations and as a guide to public health decision makers on where to focus immunization strategies [21–23]. The assays are capable of detecting asymptomatic and unreported cases of COVID-19 with a wider detection scope likely to obtain the true burden of the disease [24]. Besides, these assays depend on the humoral response to infection by production of anti- SARS-CoV-2 antibodies. Antiviral antibodies are usually detectable 10 to 14 days after exposure and are highly valuable for detecting past exposure and monitoring disease progression in a population [25].

Usually, the pre-analytical choice and quality of any analytical specimen determines the quality and reliability of laboratory results [26]. For COVID-19 RT-PCR diagnosis, nasopharyngeal swabs were primarily the specimen of choice across the globe. However, collection of these swabs is uncomfortable to the patient and poses a potential risk of contagion to the laboratory personnel [20, 21]. Further, serological assays commonly necessitate equally invasive venipuncture, while both the swabs and venous blood specimens require cold chain system for storage and transport which is often lacking in remote and underserved regions and thus limiting COVID-19 diagnosis and surveillance [27]. Dry blood spots, therefore, stand as ideal alternative specimens for SARS-CoV-2 antibody detection in such regions due to their collection simplicity, minimal invasiveness and ease of storage and transportation [28–32]. Although several sero-surveillance studies have been reported from other regions of Kenya, we are aware of only one report involving such surveillance in true pastoralist communities [16, 33–39]. The current report describes a cross-sequential survey of outpatients attending six health facilities in Kajiado County, Kenya between March, 2020 and March, 2022.

## Materials and methods

### Study site

Kajiado County is located in the southern part of Kenya, sharing an international border with the Republic of Tanzania, and is about 100 kilometers south of Nairobi, the capital city of Kenya. Based on the 2019 household census, the County has a land area of 21,900 square kilometers and a population of approximately 1,118,000 people. The County is classified under the arid and semi-arid lands (ASALs) of Kenya, which are disadvantaged in distribution of resources, infrastructure, and access to fundamental social and healthcare services. Pastoralism is the main livelihood and cultural identity of its population [40, 41].

### Study design and patient population

The present study was a descriptive study using a cross-sectional, opportunistic sampling design, and was part of a larger project that aimed to enhance laboratory diagnostics for zoonotic disease risk mitigation in underserved arid and semi-arid regions of Kenya. The inclusion criterion was patients 18 years or older that visited the facilities and gave informed consent for testing. A significant determinant of which patients were sampled at a given facility on a given day depended on case load and ability of the medical staff to obtain the sample and informed consent. We were not able to record the presenting complaints for patients from which samples were obtained, but they visited the health facilities for a myriad of reasons, most of which were not likely related to COVID-19-like disease.

### Preparation and elution of dried blood spot

Whole blood collected for other routine diagnostic testing was used to prepare duplicate blood filter paper strips (Nobuto blood sampling paper strips, Toyo Roshi Kaisha Ltd, Tokyo, Japan). The blood filter paper strips were then air-dried overnight at ambient temperature in a dust-free environment and inserted into paper envelopes that were stored at ambient temperature for variable periods (up to 4 weeks) before being taken to the Kajiado County Referral Hospital (KCRH) for storage at -20°C until they were transported to University of Nairobi Institute of Tropical and Infectious Diseases (UNITID) for analysis.

A 5 x 15 mm area of each DBS was eluted in 0.5 ml of phosphate-buffered saline containing 0.05% Tween 20 in micro centrifuge tubes by shaking at 450 rpm overnight at ambient temperature. The tubes were then centrifuged at 10,600 x g for 10 min and the eluates were transferred to other tubes and stored frozen at -20°C until assay. The eluate was thus equivalent to approximately 20 μl serum.

### Enzyme-linked immunosorbent assay

We utilized a commercial enzyme-linked immunosorbent assay (ELISA) for the detection of immunoglobulin G (IgG) against the S1 domain of SARs-CoV-2 (EUROIMMUN Quanticell SARS-CoV-2, Product ET 2606–3003, Lübec, Germany) as per the manufacturer’s instructions. Eluates from DBSs were diluted 1:101 in the sample buffer provided with the kit. One hundred microliters of the calibrator, positive, negative controls and the diluted eluates were dispensed into individual microplate wells and incubated at 37 ±1°C for one hour. An automatic microplate washer was used to wash the microplate wells 3 times using 450 μl wash buffer per well for each wash. Subsequently, 100 μl of enzyme conjugate (peroxidase-labelled anti-human IgG) was dispensed into each microplate well, followed by incubation at +37°C for 30 minutes and the wells were then washed again as described. Thereafter, 100 μl of substrate solution was dispensed into each microplate well and incubated at ambient temperature (18–25°C) for 30 minutes away from direct sunlight. The reaction was then stopped by dispensing 100 μl of stop solution into each well. Optical density from each microplate well was determined using a microplate reader at 450nm wavelength and 620nm reference wavelength.

The study adhered to the quality control measures described in the EUROIMMUN SARS-CoV-2 kit. A calibrator, positive and negative controls were included in each assay. Positive and negative control served as internal controls to determine the reliability of the test procedure. The tests were further evaluated by computing cut-off value (the upper limit of the reference range of non-infected individuals). Further semi-quantitative evaluation of the test results was done by computing a ratio of the extinction of the control or patient sample over the extinction of the calibrator. Test results whose ratio was <0.8 were considered as negative, those with ratio ≥ 0.8 to 1.1 were considered as borderline (invalid) while those whose ratio was ≥1.1 were considered as positive. All borderline test results were confirmed by retesting in a separate assay.

### Statistical analysis and variable definition

Microsoft Excel^®^ 2013 (Microsoft Corp, Redmond, WA, USA) was used to store data, and R software (version 4.1.1) was used for analysis. For categorical variables, proportions were computed. R software was used to calculate the prevalence estimates and their 95% confidence intervals using the Wilson score interval. Bivariate analysis was performed to test for associations between the independent and dependent variables, and odds ratios (OR) with 95% confidence intervals (CI) were reported. The location, gender and age were the independent variables, with the outcome variable being the SARS-CoV-2 serology test results. Variables with a P-value of < 0.05 were considered statistically significant. Binomial logistic regression was used to determine the association of study variables on SARS-CoV-2 seropositivity; patients greater than 60 years of age were used as a reference group due to their enhanced susceptibility to severe COVID disease.

### Ethical considerations

The study was approved by the Kenyatta National Hospital-University of Nairobi Ethics and Research Committee (KNH/ERC) number (P880/11/2019) and the National Commission for Science and Technology (NACOSTI) research permit number (NACOSTI/P/21/13647). All eligible patients were required to sign written informed consent before recruitment and sampling. Patients were recruited for this study beginning March 1, 2020 and ending March 31, 2022.

### Inclusivity in global research

Additional information regarding the ethical, cultural, and scientific considerations specific to inclusivity in global research is included in the S1 File.

## Results

A total of 908 participants were enrolled in the study and included in the analysis, of which 202 (22%) were males and 706 (78%) females, with an average age of 40 years (SD +15) (Table 1). The samples were collected from March 2020 to March 2022. Among the sampled participants, 223 (24.6%) were from Enkorika health facility, 58 (6.4%) were from Iltilal, 135 (14.9%) were from Mailwa, 90 (9.9%) were from Mashuru, 127 (14%) were from Meto, and 275 (30.3%) were from Mile-46. Mile-46 had the highest percentage of enrolled participants among the health facilities sampled, while Iltilal had the lowest.

**Table 1:** Characteristics of study participants (N = 908).

	Total patients tested (N)	Number test positive	Proportion positive (%)	95% CI*	P-Value
**Age (years)**					
18–40	427	19	4.4	2.8–7.0	0.00846
41–60	314	28	8.9	6.1–12.8	
≥ 60	167	19	11.4	7.2–17.4	
**Facility**					
Enkorika	223	10	4.5	2.3–8.3	
Iltilal	58	3	5.2	1.4–15.3	<0.0001
Mailwa	135	3	2.2	0.6–6.7	
Mashuru	90	14	15.6	9.1–25.1	
Meto	127	19	15.0	9.5–22.6	
Mile 46	275	17	6.2	3.8–9.9	
**Gender**					
Male	202	27	13.4	9.1–19.0	0.001036
Female	706	39	5.5	4.0–7.5	

Confidence intervals and P-values refer to testing for differences among those categories.

Overall SARS-CoV-2 seropositivity was 7.3% (66/908; 95% CI 5.7–9.1). Participant ages were divided into three categories: 18–40 years old, 41–60 years old, and >60 years old. Participants over the age of 60 had the highest proportion of seropositive cases (11.4%, 19/167), followed by those between the ages of 41–60 (8.9%, 28/314), while those between 18–40 had the lowest proportion of seropositive cases (4.4%, 19/427). Analysis of the age variable showed that participants aged between 41–60 years and between 18–40 years were less likely to have a SARS-CoV-2 seropositive test compared to those above 60 years (Table 2).

**Table 2:** Binomial logistic regression analysis of age of participants in Kajiado County and association with SARS-CoV-2 seropositivity.

Patient age	Odds ratio	95% confidence interval	P- value
18–40	0.36	0.19–0.71	0.003
41–60	0.76	0.41–1.43	0.388
>60	*Reference*	*Reference*	*Reference*

Males (13.4%, 27/202; CI: 9.1–19.0) had a higher chance of being seropositive than females (5.5%, 39/706; CI: 4.0–7.5), with gender having a significant association with seropositivity (p < 0.05).

Mashuru (15.6%, 14/90) and Meto (15%, 19/127) health facilities had the highest proportion of seropositive participants. The binomial logistic regression analysis revealed that participants from Mashuru (OR = 3.92; 1.69–9.46) and Meto (OR = 3.75; 1.72–8.66) had at least 4 times the risk of a SARS-CoV-2 seropositive result compared to those from Enkorika. Participants from the other health facilities including Iltilal, Mailwa and Mile-46 were not significantly associated with seropositivity compared to the reference location (Enkorika) (Table 3).

**Table 3:** Binomial logistic regression analysis of participants from different facilities in Kajiado County and association with SARS-Cov-2 seropositivity.

Facility	Odds ratio	95% Confidence interval	p-value
Enkorika	*Reference*	*Reference*	*Reference*
Iltilal	1.16	0.25–3.95	0.824
Mailwa	0.48	0.11–1.62	0.277
Mashuru	3.92	1.69–9.46	0.002
Meto	3.75	1.72–8.66	0.001
Mile 46	1.40	0.64–3.24	0.407

**Fig 1:**
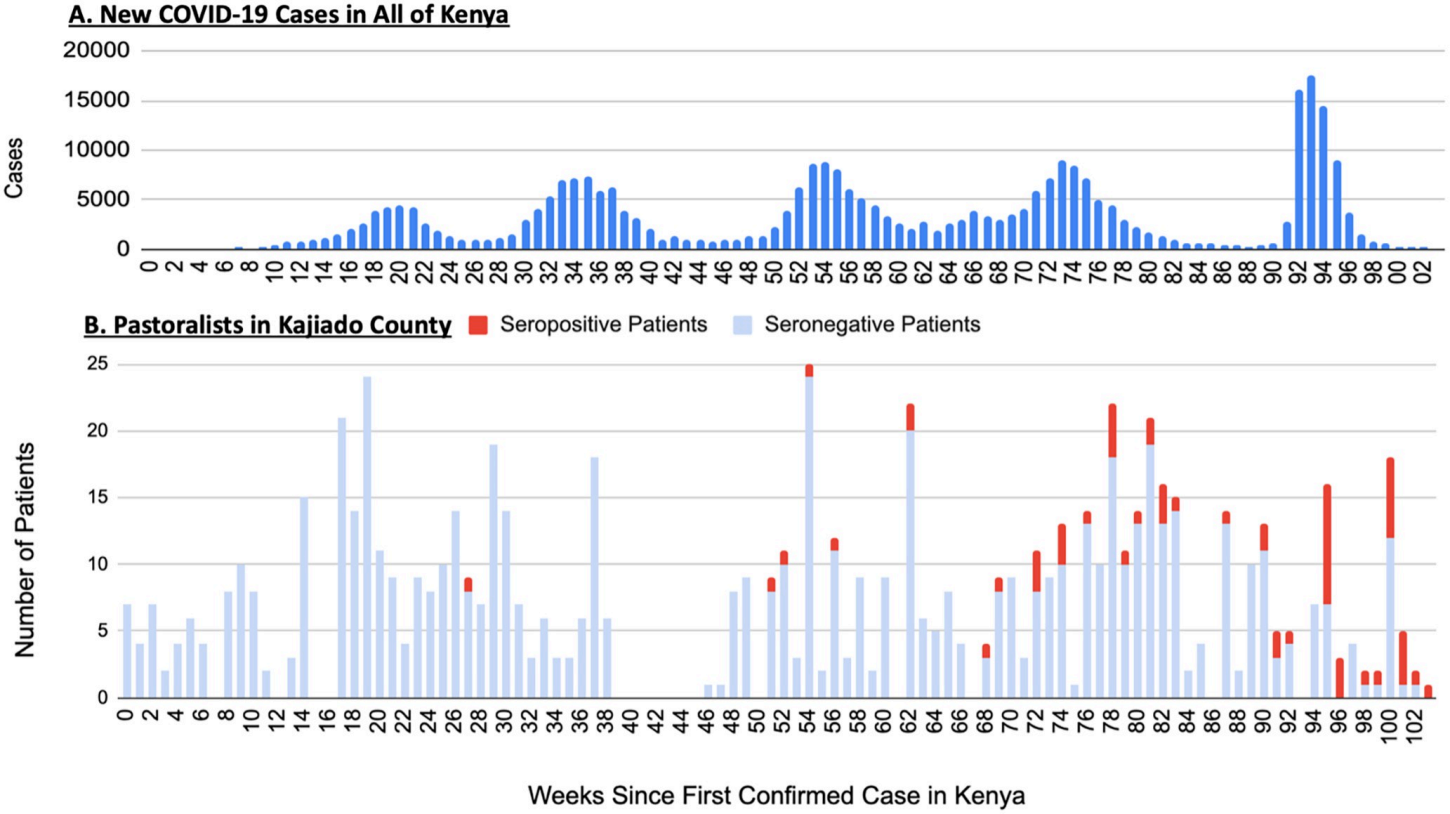
Comparison of (A) new COVID-19 cases throughout Kenya versus (B) seropositive and seronegative patients in Kajiado Country over the first 103 weeks following the first confirmed case of COVID-19 in Kenya. Data for new COVID-19 cases downloaded from Our World in Data (https://ourworldindata.org/coronavirus/country/kenya).

The samples tested were collected between March 2020 and March 2022. The first confirmed case of COVID-19 in Kenya was a traveler returning to Nairobi from the United States via the United Kingdom. Among the 908 samples we tested, a total of 66 were positive for antibodies to SARS-CoV-2. During the first year since the index patient in Kenya, only four seropositive patients were detected in Kajiado County. The remainder 62 positive samples were collected during the second year of the Kenyan epidemic (Fig 1).

## Discussion

### Principal findings

Population density and mobility were major drivers in initial SARS-CoV-2 contagion. Here we report serological evidence of SARS-CoV-2 contagion in the low-populated, remote and underserved regions of Kajiado County, Kenya [17, 42]. A large fraction of the patients sampled for this study were pastoralists who live outdoors and in low-density populations which would to some extent confer protection from transmission of respiratory viruses such as SARS-CoV-2. Moreover, soon after the first case of COVID-19 was identified, the Kenyan government implemented a travel ban that also likely mitigated exposure of pastoralists to a large number of infected patients from population centers like Nairobi. Nevertheless, with some apparent delay, the virus indeed spread to pastoralist communities as evidenced by the seroconversions we describe here. This phenomenon was also observed among pastoralist communities in Ethiopia [43].

We report an overall SARS-CoV-2 seropositivity of 7.3% (95% CI 5.7–9.1) in the six health facilities studied in Kajiado County. This was slightly higher than what was reported (5.8%, 95% CI:1.6–8.4%) in a study conducted in a refugee camp in Northeastern Kenya that utilized rapid diagnostic test serology [33]. The proportion of seropositivity in males was more than twice higher than for females, with gender being significantly associated with seropositivity. Such is consistent with many epidemiological findings in both local and international published data. The observed risk can be attributed to a high propensity to travel, increased engagement in outdoor, social and professional activities, smoking, alcoholism, poor adherence to mitigation measures, and immunological and genetic variations [44–46]. This predisposition could be higher among transhumant male pastoralists who move seasonally in search of pasture, water, and livestock markets where intermixing and increased contact among naïve and infected populations is increased [17, 47].

There was a strong association between age and seropositivity with the highest recorded among participants over 60 years of age category, followed by those between 41–60 years. This is consistent with results from a study conducted in an urban informal settlement in Nairobi, Kenya that reported significantly high seroprevalence among adults between 20–59 years [16, 48]. Similar findings were also reported in the pandemic worst stricken countries such as China, Italy, France and America attributable to an elderly-dominated population. This may correspond to waning immunity by age and vulnerability to age-related conditions such as cardiovascular disorders, hypertension, diabetes, malignancies, poor nutrition, reduced activity, and psychological distress more so in developed countries [5, 46, 49–51]. Though African countries are dominated by a younger population who are perceived to be more resistant to COVID-19 infections, poverty, poor healthcare systems and inadequate socio-economic infrastructure, antiquated cultural-religious beliefs and practices could saliently have aggravated SARS-CoV-2 transmission more so in the remote and underserved regions of the continent.

Although RT-PCR is considered the gold standard for diagnosis and surveillance of COVID-19 globally, the current study reports on serological detection of anti-SARS-CoV-2 antibodies using ELISA technique [19]. Such serologic testing provides a retrospective view of SARS-CoV-2 infection within the population and, in most cases, individuals that are antibody positive would not be expected to be actively infected and shedding virus at the time of sample collection. Since the first case of COVID-19 was confirmed, cases gradually escalated as indicated by the time series analysis. Consequently, there was an increased demand for diagnostic testing and mounting pressure on the already strained healthcare systems [52]. The utility of RT-PCR was limited due to associated high cost, requirement for high biosafety containment, specialized laboratory equipment and highly skilled personnel among others, which largely precluded its use in many health facilities especially in underserved pastoralist communities [20, 21, 52, 53]. In response, therefore, alternative diagnostic assays needed to be sought for, with different researchers recommending serological assays such as ELISA for such use [53–57]. Nonetheless, serology was not fully utilized in Kenya, with only a few studies reported in regions other than the remote and underserved regions of the country [16, 34, 35, 37–39, 48]. Only one known study among the refugees in Northeastern, Kenya was conducted in such regions that made use of rapid diagnostic test serology [33]. This study, therefore, was intended to fill this research gap.

Coupled with testing by ELISA, we used DBS sampling in place of venous blood which is considered a conventional specimen for many analytes [58]. Our study site is inhabited predominantly by pastoralist communities and falls under the arid and semi-arid regions of the country, which are mainly remote with inadequate and commonly poorly resourced and sometimes non-functional health facilities. Collection of both nasopharyngeal swabs for RT-PCR and venous blood is cumbersome and invasive with a need for experienced personnel. Further, storage and transportation of these specimens require cold chain systems which were not easily available in our study settings. Dry blood spots, therefore, became our alternative sample since they are easier to collect, store and transport with assured stability of analytes than venous blood [28, 31, 59, 60]. Evaluation and validation studies on the use of DBSs compared to venous blood samples have documented reliable results and unanimously recommended DBS sampling as an alternative to venous blood, especially in populations and regions where venipuncture is not feasible [29–31, 59, 61, 62]. Of concern is that the majority of these studies were conducted using known positive and negative COVID-19 samples rather than field clinical samples. To the best of our knowledge, this is the first COVID-19 serosurvey report that utilized DBS samples collected from a remote and underserved clinical setting in Kenya.

A relatively high seropositivity was noted in participants from two health facilities, Mashuru (15.6%, 14/90) and Meto (15%, 19/127) whose participants registered at least four times higher risk of a SARS-CoV-2 seropositive results compared to those from Enkorika. High seropositivity at Meto health facility, could have been attributed to cross-border transmission due to its proximity to the Tanzanian border through shared markets and health facilities across Kenya [62]. Tanzania experienced high transmission of SARS-CoV-2 and by the start of this study, it had not instituted any mitigation measures to curb SARS-CoV-2 transmission [63]. Mashuru location, on the other hand, is a busy trading center for both Kenyan and Tanzanian truck drivers. Truck drivers are more vulnerable to SARS-CoV-2 transmission due to increased mobility across diverse transmission zones and therefore become conduits of transmission to naive populations. A recent study in Europe, reported a 92% onset SAR-CoV-2 transmission that was attributed to increased mobility [64]. Similarly, in Kenya, a high seroprevalence of SAR-CoV-2 (42.3%) was documented among truck drivers and their assistants in Kilifi and Busia Counties [34, 39]. Enkorika health facility, seropositivity can be explained by its location, which is on the outskirts of Kajiado town. This periurban region is characterized by a mixed population and great influence from other neighbouring counties such as Nairobi which was then considered a hot spot for SARS-CoV-2 transmission. Finally, there were more female participants compared to males in this study most likely due to their higher health-seeking behavior and a positive attitude towards COVID-19 response than men [45].

Based on the time series analysis of SARS-CoV-2 transmission in Kenya and the sero-surveillance reported here, it is evident that there was very little COVID-19 activity within the pastoralist communities of Kajiado County in the first year of the epidemic and following the introduction into the country. However, during the second year, transmission inevitably increased, resulting in substantial rates of infection among both the urban and rural residents of Kajiado County.

### Conclusions

Although initial SARS-CoV-2 transmission was attributed to high population density, especially in urban centers, we report here serological evidence of SARS-CoV-2 contagion in the low-populated, remote, and underserved regions of Kajiado County, Kenya. In contrast to molecular or antigenic testing, serologic assays are typically not useful for diagnosis of current infection. However, serologic testing provides complementary information and allows more robust estimation of past transmission dynamics within a population. With the postulated need for prompt and reliable laboratory diagnosis for effective control, management, and surveillance of COVID-19 and in the face of poor health systems in remote and underserved regions, serology and DBS sampling technology are possible surrogate tools to conventional molecular diagnostic testing and venous blood sampling more importantly for large scale epidemiological screening.

### Limitations of the study

Sampling of the pastoralist population at large for this study was opportunistic or convenience rather than systematic, which could well have led to bias. The clinics and dispensaries from which patients were sampled tend to often be very busy and it is likely that the samples we obtained were collected during relatively slow periods, which could impose another sampling bias. There was a distinct gender bias in the patients from which samples were obtained and it is possible there were gender differences in intensity of exposure. Additionally, we were not able to reliably classify patients that were sampled by their presenting clinical syndrome. A final limitation of the serologic testing was that we did not assay for immunoglobulin subclasses such as IgM, which precluded detailed evaluation of the recency of infection.

## Supporting information

S1 File Inclusivity-in-global-research-questionnaire. Inclusivity in global research questionnaire.
(PDF)
